# Case Report: Anomalous origin of the right coronary artery leading to cardiac arrest induced by sexual activity: a previously unreported pathogenetic condition

**DOI:** 10.3389/fcvm.2024.1414821

**Published:** 2024-11-29

**Authors:** Junchu Tang, Da Li, Shuizhu Wang

**Affiliations:** ^1^ Department of Cardiovascular Medicine, Xinyu People’s Hospital, Xinyu, China; ^2^ Graduate School of Jiangxi Medical College, Nanchang University, Nanchang, China

**Keywords:** coronary artery anomalies, cardiac arrest, ARCA-LCS, coronary angiography, multislice computed tomography, cardiac magnetic resonance imaging

## Abstract

**Background:**

The Anomalous Origin of the Right Coronary Artery from the Left Coronary Sinus (ARCA-LCS) is a rare congenital cardiac condition where the right coronary artery emerges from the left sinus instead of the right coronary sinus of Valsalva. The clinical significance of ARCA-LCS lies in its potential to cause myocardial ischemia or sudden cardiac death, particularly under physical exertion. In this case, a patient experienced sudden cardiac arrest during sexual activity, which has not previously been reported.

**Case presentation:**

Six years ago, a 37-year-old man was admitted with sudden cardiac arrest during sexual intercourse. No previous history of hypertension or diabetes. There was no abnormality in physical examination. Transthoracic echocardiogram, bilateral carotid Doppler ultrasound, and electrocardiogram were normal. Cranial magnetic resonance imaging and magnetic resonance angiography showed no abnormalities. A treadmill exercise test revealed ischemic changes. Coronary computed tomography angiography showed ARCA-LCS, and passage through the vessel wall between the aorta and pulmonary artery.

**Conclusion:**

This case illustrates a patient with asymptomatic ARCA-LCS for 37 years who did not receive appropriate treatment during a previous visit, but who subsequently experienced a serious cardiovascular event that demonstrated the potential harm of the disease. Therefore, timely intervention in patients with ARCA-LCS, especially in high-risk groups, is critical to prevent potentially catastrophic cardiovascular events. However, in the present case report, the patient did not experience a similar event during the 6-year follow-up by avoiding overexertion and changing his lifestyle at the time of previous onset of the disease. Further studies are needed to optimize diagnostic and therapeutic strategies for ARCA-LCS.

## Introduction

Anomalous Origin of the Right Coronary Artery from the Left Coronary Sinus (ARCA-LCS) is a rare cardiac anatomical variant that belongs to a subspecies of coronary artery developmental anomalies. As reported in the literature, the two largest and most comprehensive angiography series to date, conducted by Yamanaka and Hobbs (1) over a 28-year period, enrolled a total of 126,595 patients and reported a prevalence of anomalous coronary arteries of 1.3%. Of these patients, 1,689 (1.3%) had coronary anomalies noted, and an additional 136 (0.107%) had a right coronary artery (RCA) arising from the left coronary artery sinus. Krasuski et al. (2) noted that during a retrospective analysis of 210,700 cardiac catheterizations performed over a 35-year period at a single institution, 301 adults were identified as having an anomalous coronary artery arising from the contralateral sinus of Valsalva. Interestingly, 79% of these individuals demonstrated an RCA originating from the left cusp of the aortic valve (0.11%). The results of these two large data sets suggest that there is little difference in the incidence of ARCA-LCS. Due to the heterogeneity of coronary arteries between individuals, a variety of coronary arteries abnormalities have been caused. Therefore, many classification systems are proposed for different classification criteria, including classification systems based on different dimensions, such as anatomical structure, function, or clinical significance (3, 4). As a type of coronary artery origin anomaly (or ACAOS), the prevalence of ARCA disease is estimated to be 0.92% based on angiographic data (5). To date, its incidental detection is commonly observed during coronary angiography (CAG) or other cardiac imaging procedures. Of course, the evolution of non-invasive imaging technologies, specifically the utilization of multislice computed tomography (MSCT) and cardiac magnetic resonance imaging (MRI), has assumed a pivotal role in facilitating a definitive diagnosis.

After the diagnosis of the disease, the choice of treatment requires careful consideration. This anomalous course can cause external compression, sharp angulation, or twisting (torsion) of the artery, particularly during physical exertion when oxygen demand is increased, leading to compromised myocardial perfusion. If not surgically corrected, these factors can result in myocardial ischemia, which may progress to myocardial infarction, life-threatening arrhythmias, or sudden cardiac deaths (6). Therefore, timely diagnosis and treatment are crucial for these patients. However, the question of whether surgical treatment should be undertaken when a patient is diagnosed with ARCA-LCS disease requires careful consideration. The clinical outcome for ARCA is most often benign (7). Therefore, is surgery needed for every patient and does a more optimal treatment strategy exist for cases where the disease prevalence is low and the majority of patients are asymptomatic?

## Case report

**Figure 1:**
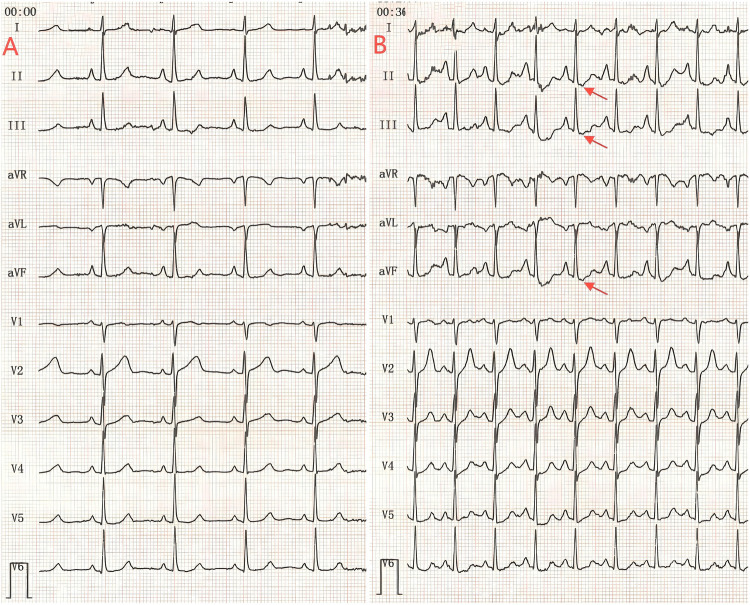
**(A)** The ECG recorded at rest, showing baseline readings. **(B)** Captured during the recovery phase after exercise, leads II, III, and aVF exhibit a noticeable ST-segment depression (red arrow). This finding suggests ischemic changes in these inferior leads, likely provoked by physical exertion.

**Figure 2:**
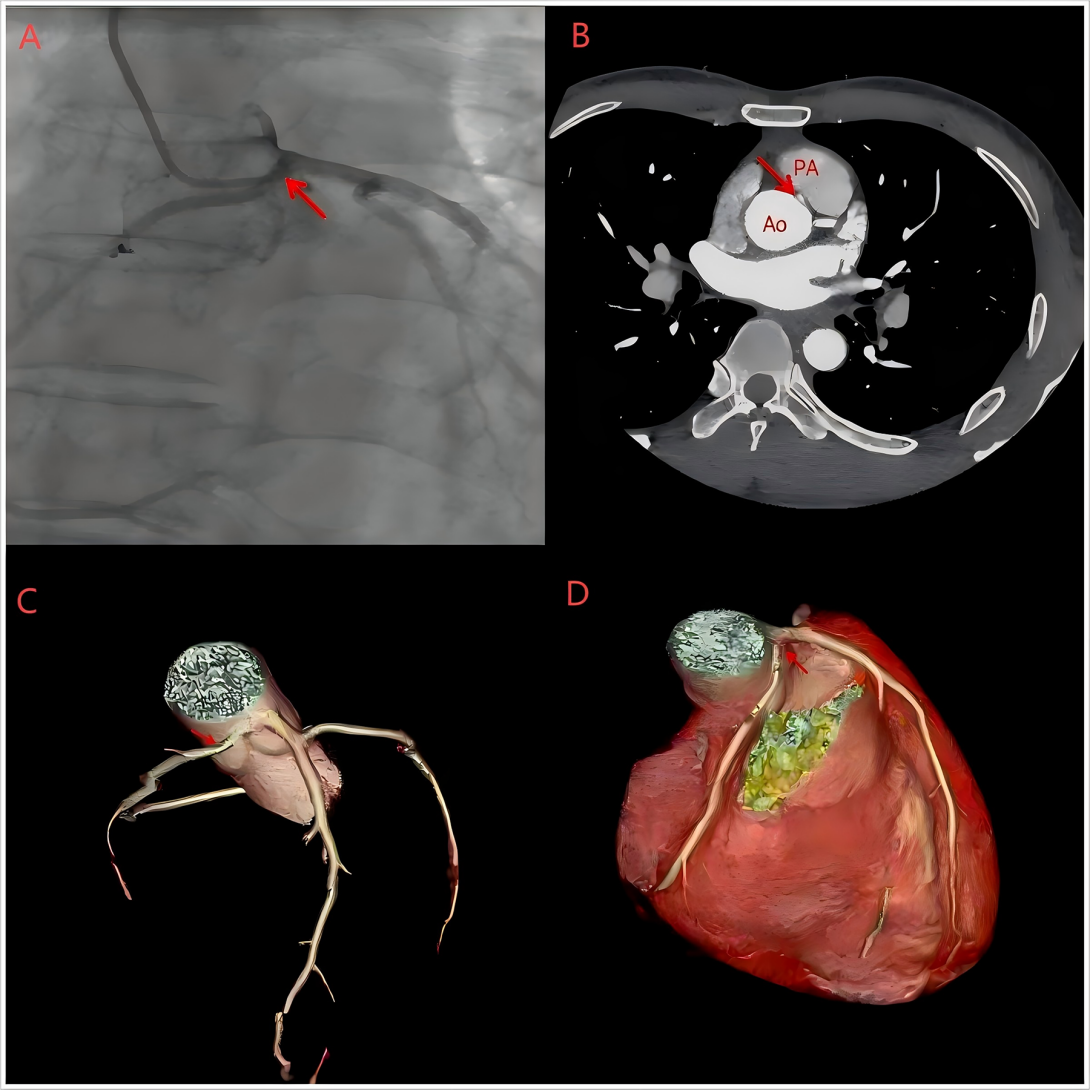
**(A)** CAG revealed a common ostium (red arrow) that produced the right coronary artery and the left coronary artery. **(B)** CCTA shows the proximal region of the right coronary artery located between the aorta and the pulmonary artery. **(C,D)** Three-dimensional reconstruction of the coronary artery showing the abnormal origin of the RCA, with its branches crossing the left main trunk at an acute angle. In addition, the RCA runs between the aorta and the pulmonary artery, and its proximal part of the diameter is slightly reduced.

Six years ago, a 37-year-old man with a history of vertigo spanning over a decade experienced sudden cardiac arrest during sexual activity. He had no previous history of hypertension or diabetes. According to his wife, he lost consciousness immediately, which lasted for approximately 10–20 s before resolving spontaneously. He was then urgently taken to the hospital by his family. The patient was a habitual smoker and had a known allergy to alcohol. After hospitalization, the patient's physical examination (PE) revealed no obvious clinical signs and no heart murmur was heard. No abnormalities were identified in the patient's medical examination. Electrocardiogram (ECG) confirmed a sinus rhythm. Bilateral carotid ultrasound showed no significant stenosis or plaque formation. Transthoracic echocardiogram revealed an ejection fraction of 60% with normal ventricular wall motion and no evidence of hypertrophic cardiomyopathy. Laboratory tests such as blood routine, liver and kidney function, electrolytes, blood glucose and lipid, myocardial enzyme profile, troponin, and coagulation function did not reveal any significant results. To determine the underlying cause of a condition or disease, the necessary tests must be performed to clearly identify the etiological factors. We conducted a series of examinations on the patient, including MRI and magnetic resonance angiography (MRA) of the brain, to assess neurological status. The results indicated that there were no significant abnormalities in the intracranial soft tissue or vasculature. During the treadmill exercise test (TET), the patient's ST-segment depression was recorded in leads II, III, and aVF (approximately 0.2–0.3 mV), suggesting possible myocardial ischemia (Figure 1), but no chest pain or syncope was observed in the patient during the TET. Due to insufficient physical endurance, the test was ultimately terminated. The TET reached a level classified as stage II (submaximal exercise), indicating that the patient's exercise tolerance was within normal limits; however, further evaluation of cardiovascular health is warranted. During CAG, the patient's RCA may have an abnormal origin due to the inability to selectively engage the vessel during selective right coronary angiography (Figure 2A). With the patient's consent, we proceeded with coronary computed tomography angiography (CCTA) to further assess his condition. The CCTA results revealed an anomalous origin of RCA, which courses between the aorta and pulmonary artery (Figure 2B). In addition, the ARCA forms an acute angle with the left main artery, with mild proximal narrowing (Figures 2C,D). Given the patient's symptoms after exertion and this anatomical variation, surgical intervention was recommended; however, the patient opted for conservative management. We advised him to prioritize rest, avoid strenuous activities, and adjust his sexual lifestyle if necessary. The patient did not experience anything resembling cardiac arrest during a recent follow-up.

## Discussion

The most common congenital heart lesions associated with sudden death during exercise include hypertrophic cardiomyopathy, coronary artery anomalies, Marfan's syndrome, and aortic valve disease (8–10). Most coronary artery abnormalities do not produce any symptoms, but some patients may experience adverse events, such as arrhythmia, angina, syncope, myocardial infarction, and even sudden death, with an incidence of up to 33% (11, 12). Even though ARCA-LCS is a rare coronary artery anomaly, the diagnosis of this disease is essential to prevent possible cardiovascular complications. In 2000, Basso et al. summarized the left and right coronary arteries originating from the wrong aortic sinus based on the clinical characteristics of the patients and identification of clinical markers (13). Today, CAG and MSCT play a key role in the diagnostic process of targeting ARCA-LCS. In these patients, MSCT was able to provide more accurate three-dimensional vascular images than conventional angiographic techniques, thereby facilitating the identification of high-risk alignments of the coronary arteries between the pulmonary arteries and the aorta (14). With the increasing sophistication of imaging equipment, there is a need to think about and understand this issue in depth and to recognize that MSCT can more accurately diagnose patients with ARCA-LCS. In addition, recent studies have identified myocardial scarring in regions supplied by coronary arteries with anomalous origins in sudden cardiac death cases involving individuals with prior R-ACAOS, as observed in autopsies. This underscores the importance of using cardiac MRI (CMRI) with late gadolinium enhancement (LGE) to assess both the prevalence and clinical implications of these scars in living patients (15).

Nowadays, pathophysiological studies have revealed that the onset of symptoms of such diseases stems from ischemia triggered by insufficient oxygen supply to the myocardium (16). This may be attributable to numerous surgically correctable anatomic factors (17, 18), including the interarterial path of the anomalous coronary artery, the morphology of the anomalous coronary artery lumen (i.e., round, slit), the angle of origin, the intramural course, and the length of the wall. In addition, in the case of R-ACAOS, which travels between arteries, it leads to a significantly higher incidence of atherosclerosis-related symptoms and events (19). After analyzing the imaging findings of this patient, the following features were identified, including the presence of coronary arteries of abnormal origin traveling between the aorta and the pulmonary artery, emanating from the aorta at an acute angle, with a slit-like pattern of openings, and the presence of intramural segments of coronary arteries, as well as their length. For this reason, the main goal of surgery in this disease is to reduce the risk of ischemia and to graft the abnormal coronary arteries to a suitable location. If feasible, coronary unroofing is typically favored in patients with an early intramural course. Alternatively, coronary reimplantation, fenestration, neo-ostia formation, or a combination of these techniques may be employed to provide additional treatment options (20). For patients with this type of disease who fail to undergo surgical treatment, some restrictive activity measures may be required. The American Heart Association/American College of Cardiology guidelines have clarified the limitations of exercise for anomalous coronary artery origins. These are described under the heading “Anomalous Coronary Arteries from the Contralateral Coronary Sinus” (21). Recently, Sajjadieh Khajouei et al. showed that interarterial traveled R-ACAOS resulted in significantly higher rates of atherosclerosis-related symptoms and events compared to other types of RCA abnormalities, and coronary intervention significantly improved cardiac function class irrespective of R-ACAOS category (19).

A great deal of research has been carried out on the causes of symptoms in ARCA-LCS; however, to date, there has not been an absolute key factor, and Taylor et al. suggest that the slit pattern of the coronary artery openings and the stenosis of the distal intramural arteries may be the most important factors (22). In the future, by deepening our understanding of the pathophysiology and etiology of this rare coronary artery anomaly and by improving diagnostic and therapeutic strategies, we expect to provide better clinical management and improved prognosis for patients affected by it. In the meantime, a large-scale, multicenter prospective study is necessary to obtain more epidemiological data on this rare coronary artery anomaly and its therapeutic effects.

## Conclusion

RCA originating in the left coronary sinus is a rare anomaly that can lead to serious cardiovascular complications in some patients due to the possibility of arterial compression or abnormal alignment during exertion. However, in cases such as ours, conservative treatment focusing on changing sexual lifestyles and reducing exertion is effective in preventing symptom recurrence and adverse events. This highlights the value of an individualized approach to treatment, as in the past it was thought that asymptomatic or low-risk patients could benefit from non-surgical interventions, whereas patients with symptoms or evidence of ischemia could be treated with interventional strategies. In any case, in patients with R-ACAOS, a comprehensive analysis is necessary to further define management and treatment strategies for this disease.

## Data Availability

The original contributions presented in the study are included in the article/Supplementary Material, further inquiries can be directed to the corresponding author.
